# The *Last Aid Course Professional* as a Low-Threshold Opportunity for Professionals from Health and Social Care to Talk About Dying, Death and Grief and to Learn the Foundations of Palliative Care—A Mixed-Methods Study

**DOI:** 10.3390/healthcare14030401

**Published:** 2026-02-05

**Authors:** Georg Bollig, Boris Knopf, Dirk Aumann, Marina Schmidt, Raymond Voltz

**Affiliations:** 1 Department of Anesthesiology, Intensive Care, Palliative Medicine and Pain Therapy, Helios Klinikum Schleswig, 24837 Schleswig, Germany; 2 Department of Palliative Medicine, Faculty of Medicine and University Hospital, University of Cologne, 50924 Cologne, Germany; raymond.voltz@uk-koeln.de; 3 Last Aid Research Group International (LARGI), 24837 Schleswig, Germany; 4 Letzte Hilfe Deutschland gGmbH, 24837 Schleswig, Germany; boris.knopf@letztehilfe.info (B.K.); dirk.aumann@praecaveo.de (D.A.); marina.schmidt@letztehilfe.info (M.S.)

**Keywords:** Last Aid Course Professional, death, dying, death literacy, grief literacy, palliative care, end-of-life care, education, curriculum, healthcare, multi-professional, physicians, nurses, paramedics, mixed-methods

## Abstract

Background: Last Aid Courses (LACs) for the public aim to enhance the public discussion about dying, death and grief and to increase the awareness for palliative care throughout the whole society. Based on the wishes and needs of professionals from health and social care and results from previous studies on LACs, a longer Last Aid Course Professional (LACP) was developed. The aim of the present study was to evaluate the experiences and views of course participants and instructors on the Last Aid Course Professional (LACP) with ten teaching hours including the foundations of palliative care based on the storyline method. Methods: A mixed-methods approach was used including qualitative and quantitative data from a questionnaire for LACP participants and focus group interviews of LACP instructors. Results: A total of 394 of the 422 participants participated in the study by returning a questionnaire (response rate 93%). The age ranged from 21 to 81 years (median 45 years). In addition, 14 instructors participated in two focus group interviews. The results from the questionnaires showed that 84% of all participants assess the course as useful for all professionals working in health and social care. The qualitative data show that the LACP was well accepted by different organizations and participants from different professions. The participants welcome the opportunity for interprofessional exchange and the possibility for a change in perspective, as well as getting insight from different perspectives, reflecting on ethical challenges, and working on different options for action in palliative care. Lack of staff was the main barrier for participation in the LACP. Conclusions: The LACP is very well accepted by the participants and is a good option for palliative care education for professionals from health and social care.

## 1. Introduction

The main aim of the Last Aid Course (LAC) for the public is to increase both death and grief literacy; to improve the dialogue about serious illness, dying, death and grief throughout the whole society; and to inform everyone about palliative care options in the community. In addition, the LAC aims to empower everyone to participate in palliative care provision in their community. The Last Aid Course for the public consists of four modules that are 45 min each. It is part of the public knowledge approach to palliative care, first described by Bollig in 2008, which aims to spread knowledge about dying, death, grief and palliative care in the whole society [1]. LACs for adults were introduced in Germany in 2015 and Last Aid Courses for kids and teens from 7 to 17 years started in 2018 [1,2,3,4]. The LAC is a widely used approach to Public Palliative Care Education (PPCE). At present, LACs have been introduced in 23 countries in Europe, Australia, Canada, Brazil and Singapore. The experiences with LACs from different countries are in general very positive. A number of scientific articles and book chapters on Last Aid have been published [1,2,3,4,5,6]. Many children, teenagers and adults want to talk about death and dying and to learn to care for seriously ill and dying people [1,2,3,4,5]. Many LAC participants state after participation in an LAC that they feel empowered to engage in palliative care in the future and that they would recommend the course to others [2,3,4].

In a multi-centre study on Last Aid Courses for the public, it was shown that 9.4% of the 5469 participants had a medical profession as a professional background and many of these participants were doctors or nurses [3]. The introduction of an LAC for all employees of a German University Hospital revealed that nurses and physicians would prefer a slightly extended LAC that is more tailored to suit the needs of healthcare professionals [5]. These needs included an expanded curriculum with more time for the discussion of cases, ethics and a level of information adapted to professionals rather than lay people [5]. Thus, a low-threshold opportunity to learn about the foundation and the most important aspects of palliative care for healthcare personnel and other professions in community health and social care was needed to provide a basic introduction to palliative care and hospice philosophy and to provide a space to reflect on one’s own attitudes towards serious illness, dying, death and grief. These findings from scientific research on LACs led to the formation of a working group of Letzte Hilfe Deutschland gGmbH [6] and a consensus process on a curriculum for a Last Aid Course Professional (LACP). Based on the wishes and needs of professionals in health and social care and results from previous studies on LACs, a longer Last Aid Course Professional (LACP) was developed. The LACP was pilot tested as an online course during the COVID-19 pandemic because classroom teaching was not possible due to existing meeting restrictions. The results of the pilot study indicated that the LACP was very well accepted by healthcare professionals as a space to discuss dying, death and grief in general, to reflect on one’s own attitudes and to learn the basic principles of general palliative care and hospice philosophy [7]. The article on the LACP pilot study [7] received an award for the best scientific article in the German Journal of Palliative Medicine in the year 2023. In the context of this article, the terms hospice philosophy and palliative care are used for the philosophical underpinnings and practice of patient- and family-centred care for terminally ill patients in order to improve quality of life and to enable a “good death” as far as possible based on the ideas of Dame Cicely Saunders [8].

### Aims of the Study

The main aim of the current study was to explore the experiences and views of the Last Aid Course Professional participants on the course and and its contents as well as the experiences of the instructors with the new course format and their observations from teaching professionals from different healthcare professions, social services, and those who care for the elderly and work with disabled people.

## 2. Materials and Methods

### 2.1. The Concept and Curriculum of the Last Aid Course Professional (LACP)

**Figure 1:**
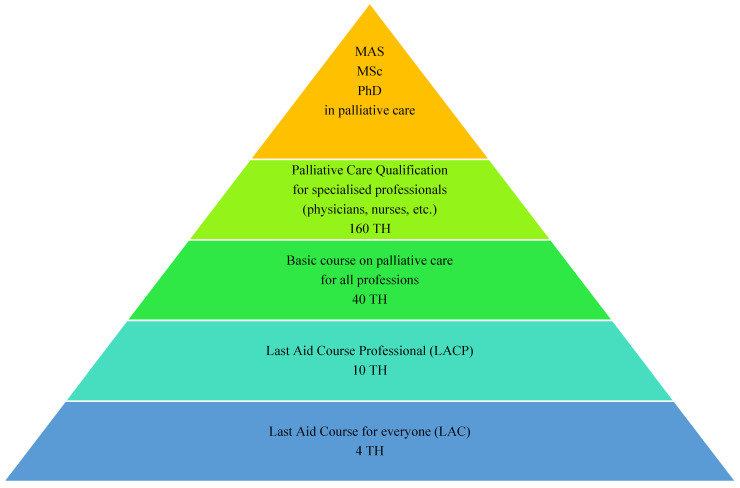
Different course formats for learning palliative care in Germany. (TH = Teaching Hours of 45 min; MAS = Master of Advanced Studies; MSc = Master of Science; PhD = Doctor of Philosophy).

Based on the experience of many medical professionals who have participated in LACs for the public and the scientific evidence suggesting that nurses and physicians in particular would like an extended Last Aid Course for professionals [3,5], the German non-profit non-governmental organization (NGO) Letzte Hilfe Deutschland gGmbH from Schleswig established a working group consisting of experts from the fields of palliative care and education in order to find a consensus on a methodological approach, curriculum and the contents of a Last Aid Course Professional [6,7]. The working group consisted of experts with different professional backgrounds and expertise and included a variety of different fields such as medicine, nursing (working in elderly care and specialized palliative care), hospice care, teaching, sociology, counselling and the emergency medical service (EMS). The working group had one meeting in person and seven online meetings. All members of the working group agreed on the final curriculum and the course contents. The course is usually held on one single day with 10 teaching hours (each 45 min) plus breaks. Course contents include a short introduction to hospice philosophy and basics of hospice and palliative care; reflection on the participants’ personal attitudes towards death and dying; and understanding of the different views and tasks of different professions, as well as multidisciplinary perspectives on medical care and palliative care in general and the need for networking in palliative care and a look into existing networks and cooperation partners in the local community and region. The LACP was designed as a connecting link between the LAC for the public and the specialized training courses in palliative care for professionals with 40 to 160 teaching hours that already exist in Germany and other countries. Figure 1 shows the different course formats that are available for learning palliative care in Germany.

The LACP provides minimal basic knowledge about palliative care for a broad range of professionals and was thus meant to serve as a sort of appetizer to interest professionals to engage in more specialized education in palliative care. Therefore, the participants receive information about existing possibilities for further palliative care education during the course. The course topics are introduced based on the storyline method [9,10] that highlights the challenges and options of palliative care, for example in a nursing home setting, with cases that involve different professionals including nursing home staff, the general practitioner, paramedics, etc., as well as the relatives. The participants are encouraged to examine a role that is different to their usual professional role. Course participants work in groups on the various stages of a disease and the dying process in a constructed case. The results of the working groups are then presented by members of the different groups and discussed in class. After these presentations and discussions, a lecture on the included topics is provided by one of the two instructors who lead the LACP. This lecture does provide background information on the topic of the group discussion, such as decision-making, ethics, and appropriate level of medical treatment, and also helps to clarify questions that are raised during the group discussions. In addition to the lectures and case discussions, reflection on the participants’ own attitudes towards death and dying is encouraged and discussed in the whole group. The reflections include the participants’ own mortality and discussion of different perceptions on quality of life, preferences for end-of-life care, and advance care planning (ACP) of the caregivers and significant others. Important topics of the course curriculum are preserving dignity and dignity-conserving care according to Chochinov [11] and ACP, as well as ethical and practical aspects of palliative care in different situations and surroundings. The included case discussions enhance the multidisciplinary and interdisciplinary discussion and enable the participants to change their perspective and to use meta-positions. In contrast to the LAC, where the focus is the perspective of the participants, who can be informal carers, people in need of palliative care or people who are interested to learn about palliative care, the focus of the LACP is on the professionals’ different perspectives and their reflections based on both professional and personal standpoints. Table 1 provides an overview of the contents of the four modules of the LACP.

There are some minor modifications to the LACP as an online course (OLACP). These include suggestions for the modification of the introduction round and the group work on the cases in online breakout sessions. The LACP instructors all have experience in the field of palliative care and teaching, are active instructors for the LAC and have participated in an additional instructor course lasting 1.5 days in order to be certified as LACP instructors. All course participants receive a short summary after the course. This summary includes information about the covered topics and provides information and sources for further learning and options for further palliative care education opportunities (see Figure 1).

### 2.2. Setting and Participants

The LACP is usually held during one day with 10 teaching hours (45 min each). Similarly to the LAC for the public, it consists of 4 modules with standardized contents (see Table 1). The LACP courses that were included in the current study were held in the German federal state of Schleswig-Holstein and were supported and in part funded by the Ministry for Social Affairs, Health, Youth, Family and Elderly of Schleswig-Holstein. Professionals from health and social care from different contexts such as hospitals, nursing homes, facilities for the disabled, doctors’ offices, emergency medical services and community healthcare were invited to participate. Although the target group of the course was professionals from health and social care, participants from other professions were allowed to participate. Information about the course was spread through the media, internet and by written or oral invitation by a member of the project team and the German NGO Letzte Hilfe Deutschland gGmbH via email or telephone.

### 2.3. Data Collection and Analysis

The study was based on a mixed-methods methodology using a combination of quantitative and qualitative data [12,13,14,15,16]. A sequential explanatory mixed-methods study design was used. It started with quantitative data from a questionnaire and then used qualitative data to explain the numerical findings. All LACP participants received a questionnaire with quantitative and qualitative questions about their views and experiences of the LACP. All participants gave their informed consent to participate in the study. The questionnaire for the participants is shown in Table 2. All LACP participants were informed about the purpose of the study and were asked to provide their feedback using a paper questionnaire. The used sampling strategy in our study was to include every LACP participant who provided informed consent to participate during the study period. All participants were informed that returning the questionnaire was voluntary and that participation in the study was voluntary. The questionnaire was distributed during the course. Quantitative data collected from the questionnaire were analyzed and described by quantitative description. In addition to the questionnaire for the course participants, in the second step of the data collection, LACP instructors were invited to participate in focus group interviews via Zoom. The focus group interviews were recorded digitally and transcribed verbatim with assistance from amberscript [17]. The reason for using mixed-methods in the study was to provide a rich and thick description of the investigated topic. Data analysis of the qualitative data was based on qualitative descriptions and an inductive coding approach [14,15,16]. The focus group interviews via Zoom were led by an experienced researcher with extensive experience in qualitative research. In the focus group interviews, a semi-structured guide with the following questions was used to facilitate the group discussion:

- What are your views on and experiences with the Last Aid Course Professional?
- What are your positive experiences with the LACP?
- What are your negative experiences with the LACP?
- What is your experience with the organization of the LACP and recruitment of participants?
- How can the change in perspective be facilitated in the Last Aid Course Professional?
- What are your experiences with the group work in the LACP?
- What would you like to change in the LACP?
- Do you have suggestions for additional content that should be included in the LACP?

The first question was used as opening question. The other questions from the interview guide were used when appropriate and were skipped if the theme had already been mentioned or brought up by the focus group participants. During the focus group interview, the researcher asked follow-up questions and clarifying questions in connection to the topics that were brought up by the participants. The focus group interviews were held online with the software Zoom and the video and audio files were stored digitally.

The questionnaires were distributed to the participants and collected on paper, scanned and stored electronically. The period for data collection lasted from May 2022 to September 2024. All paper questionnaires were scanned and stored electronically on a laptop after collection. Access to the research data was limited to the researchers who are authors of the current paper. Personal data that were collected included profession, age and gender. The informants had the choice to provide personal data or to answer the questions without providing personal data. Qualitative description was used to provide straightforward descriptions in everyday terms of the participants’ and instructors’ views and experiences from the focus group discussions and the open questions from the questionnaire [14,15,16]. The methods used to analyze the qualitative data were qualitative description and qualitative content analysis [13,14,15,16]. Analysis of the qualitative data was performed by GB and BK by repeated readings of the transcripts and establishing preliminary codes from the data material. The preliminary codes from the first round of analysis were discussed by the research team and revised in two more discussion rounds. As a final step of the qualitative data analysis, the findings were questioned by using meta-positions [13]. GB, BK and the co-authors participated in the analysis process of the qualitative data. All authors have experience in the analysis of qualitative data. After all authors agreed both on the codes and themes that derived from the qualitative data material, the analysis process was finished. In addition to the data from the questionnaires and interviews, some of the researchers used field notes made during the interviews. These field notes included descriptions and preliminary interpretations that served as a control measure to question the themes that emerged from the data material. All steps of the study, including data collection, analysis, and presentation of the qualitative data, were based on the Consolidated criteria for reporting qualitative research guidelines (COREQ) [18]. In order to ensure rigour, reliability and validity of the presented research, we followed the recommendations of Mays and Pope [19]. These included, e.g., the used sampling strategy to include all participants who provided informed consent to participate during the whole study period; ensuring the reliability of the analysis process by coding by different researchers and a discussion of the preliminary and final codes by the research group; seeking validity through source triangulation using LACP participants and instructors as informants in the data collection; and using meta-positions to minimize researcher bias in the analysis and interpretation of the data [19].

**Table 2:** Last Aid Course Professional questionnaire.

**1.** **Evaluation of the course content**						
(Assessment: 1 = very good, 2 = good, 3 = satisfactory, 4 = sufficient, 5 = insufficient, 6 = poor) Please make one cross for every module and one for the whole course						
**Topic**						
	**1**	**2**	**3**	**4**	**5**	**6**
1. Dying as a normal part of life						
2. Planning ahead						
3. Relieving sufferring and symptom management						
4. Final goodbyes						
**Assessment of the whole course**						
**2. Your personal impression** (Please write)						
a. This topic was most interesting for me…						
b. I have gained confidence in dealing with…						
c. I did not like …						
**3. Your personal impression** (Please make a cross for yes and no)						
a. The course is helpful for all professions within healthcare/care	□ yes		□ no			
b. I have learned many new things	□ yes		□ no			
c. The contents have been taught in an understandable way	□ yes		□ no			
d. I will recommend the course to others	□ yes		□ no			
**4. Which course format do you prefer?**	□ Online		□ Classroom teaching in person			
**5. Profession:**	**Age:**					
**Gender (female/male/other):**						
**6. Please use this space for other comments or suggestions** (You may use the back side too)						
**Thank you very much for your kind help!**						

### 2.4. Ethical Considerations and Ethics Approval

The current study is part of a large long-term research project on Last Aid Courses that has been reported to the Regional Ethical Committee of Southern Denmark and was started in 2018 (The Regional Committees on Health Research Ethics for Southern Denmark; nr. 20182000-33). As data for the current study were collected in Germany, a second ethics approval was also sought in Germany from the ethics committee of the Europäische Fachhochschule Rhein/Erft (EUFH) Köln (Ethics committee of the CBS University of Applied Sciences Cologne; nr. 250418). In order to protect the privacy of the participants, only the following personal data were collected: profession, age and gender. The informants had the free choice to participate in the study and were free to provide the above-mentioned personal information or not without a risk for consequences for them. Informed consent was obtained from all participants. All participants received information about the purpose of the study prior to participation. All participants had the possibility to ask questions prior to consenting to participate in the study. The LACP participants in the study were asked to complete a questionnaire after participation in the course. LACP instructors were invited to participate in focus group discussions via Zoom. The study described in this article is part of a large research project led by the Last Aid Research Group International (LARGI) that evaluates the effects of Last Aid Courses.

## 3. Results

The project period lasted from 1 July 2021 to 31 July 2024. The results are presented separately for the quantitative and qualitative data from the questionnaires from LACP participants, the qualitative data from focus group interviews of LACP instructors and the experiences from implementation in the German federal state of Schleswig-Holstein.

### 3.1. Results from the Quantitative Data from the Questionnaires for LACP Participants

Within the project period between 1 July 2021 and 31 July 2024, a total number of 42 LACPs were planned. Of these, 30 LACPs with 422 participants were held (4 in 2022, 19 in 2023 and 7 in 2024). Twelve planned courses had to be cancelled due to a lack of particpants. The main reason for cancellation was lack of personnel in patient care, which made course participation impossible for many professionals. The included LACPs had a minimum of 6 and maximum of 22 participants. A total of 394 of the 422 participants participated in the study by returning a questionnaire, resulting in a response rate of 93%.

**Figure 2:**
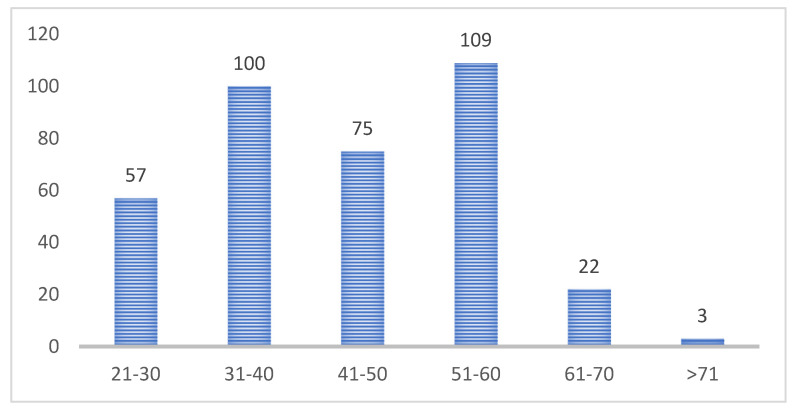
Age groups of the LACP participants (age in years; *n* = 366).

**Figure 3:**
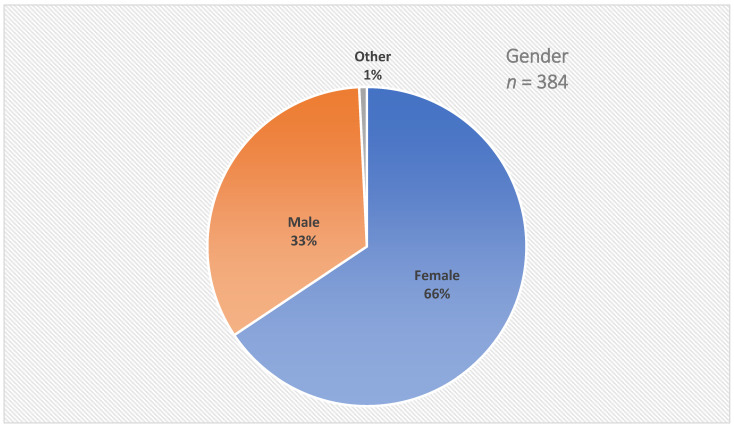
Gender identity of the LACP participants.

A total of 366 informants provided information about their age. The age of the participants varied between 21 and 81 years; the median was 45 years. A total of 22 participants were between 61 and 70 years old, and three participants were 71 or older. The age groups of the LACP participants can be seen in Figure 2. Figure 3 shows the gender identity of the LACP participants.

A total of 384 participants provided information about their gender identity: 252 were female, 129 male and 3 identified as other.

A total of 373 of the 394 informants (95%) provided information about their profession. Table 3 shows a list of the different professions of the LACP participants. Most of them were from nursing (*n* = 113, 29%), emergency medical service (*n* = 96, 24%) and elderly care (*n* = 45, 11%) contexts. Nursing and elderly care combined were the largest participant group (*n* = 158, 40%).

A total of 365 of the 394 informants stated their preference for the course format. Most participants preferred courses in person and only 6.6% preferred online courses. Although the target group of the course are professionals from health and social care, participants from other professions were allowed to participate.

**Figure 4:**
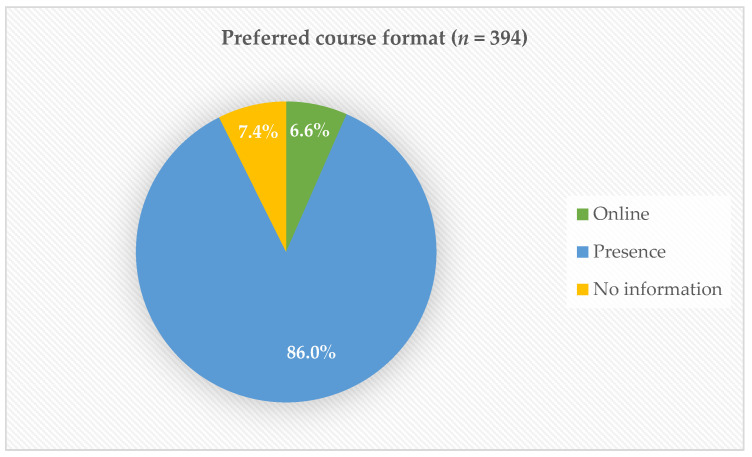
Preferred course format: online vs. in person.

Figure 4 shows the preferred course format of the participants. Table 4. Illustrates the ssessment of the LACP modules by the participants (*n* = 394).

All informants were asked to judge the course contents by using one of the following characters: 1 = very good, 2 = good, 3 = satisfactory, 4 = sufficient, 5 = insufficient, 6 = poor.

More than 80% of the informants assessed all modules as very good or good and 80% judged the whole course with the characters for very good or good.

In the subgroup of emergency medical service personnel (*n* = 96), 64% of the informants assessed all modules as very good or good and 91% judged the whole course with the characters for very good, good or satisfactory. In the subgroup of educators and special education nurses, 88% of the informants assessed all modules as very good or good.

In addition the informants were asked to answer some questions with yes or no. Their answers are shown in Table 5.

The comparison of the results of the three groups provided in Table 5 shows that 98% or more found that the course contents had been taught in an understandable way. Interestingly, the groups showed differences in the percentage for recommending the LACP to others, ranging from EMS personnel with 74% to educators and special education nurses with 96%. The results suggest that the LACP in its current form is highly appreciated by educators and special education nurses (100% think that the LACP is helpful for all professions within healthcare and care), whereas EMS personnel in general are more sceptical (55% think that the LACP is helpful for all professions within healthcare and care).

### 3.2. Results from the Qualitative Data from the Questionnaire for LACP Participants

The qualitative part of the questionnaire for LACP participants included questions about the informants’ personal impressions and comments on the following topics:

- Which topic did I particularly like?
- I gained confidence in dealing with …
- What I did not like:
- Additional comments or suggestions

The informants’ responses were open-ended. Some participants responded with keywords only, whereas others provided detailed written feedback.

*Which topic did I particularly like?*

Many participants expressed overall satisfaction with the course. Common answers included:

*Everything was good.*

*All topics were interesting.*

The content met the participants’ need for information and was presented in a well-structured manner. Group work and the use of the storyline method were especially appreciated. The practical learning was connected with case discussions about Mrs. Schulz, a fictional case from long-term care home that was developed for teams with different roles. Working on case studies encouraged perspective-taking and provided insight into other professions. Topics such as autonomy, communication, saying goodbye, and symptom relief were seen as particularly helpful:

*New practical tips for symptom relief—a great way to expand your toolbox.*

Many valued the reflection on personal attitudes and mortality.

*I gained confidence in dealing with…*

Many participants reported gaining confidence in dealing with dying individuals, their relatives, and their own advance care planning:

*Dealing with dying people, their relatives, and also thinking about my own approach and planning.*

This included both attitudes and practical aspects such as mouth care. Participants from the emergency services also reported increased confidence in palliative contexts. Some highlighted the importance of involving palliative care early and discussing end-of-life wishes:

*Start conversations about advance directives and planning. Involve the palliative care team early on.*

Trust in one’s own gut feeling was also reported to be strengthened.

*What I did not like:*

Criticism focused primarily on the course’s duration.

*Too much input for one day.*

From the perspective of some emergency responders, criticism primarily focused on the limited relevance to their daily practice:

*Not enough focus on emergency services.*

Some participants would like to have more content on sudden death, dying at a younger age, and legal aspects.

*Additional comments or suggestions:*

Most participants were very satisfied with the overall course design. Some suggested making the course mandatory for all healthcare professionals:

*This course should be compulsory for everyone (working in healthcare).*

Interdisciplinary exchange was seen as especially valuable. Many participants called for more networking opportunities, additional modules (e.g., communication skills and role play), and greater involvement of leadership:

*A very important course for all leaders.*

### 3.3. Results from the Focus Group Interviews of LACP Instructors

**Figure 5:**
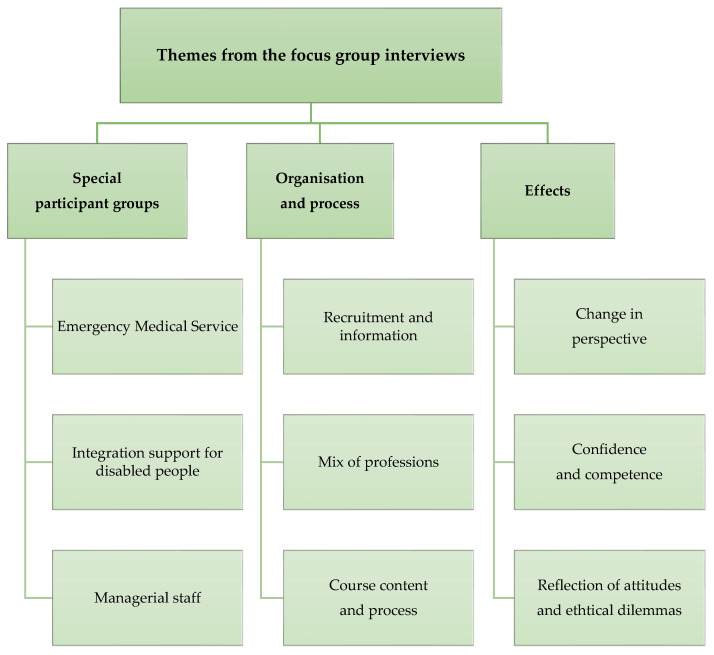
Themes from the focus group interviews of LACP instructors.

Fourteen LACP instructors participated in two focus group interviews via video. Within the period for data collection from May 2022 to September 2024, the focus group interviews were recorded in December 2023 and September 2024 to collect the experiences of the LACP instructors. The analysis of the data from the focus group interviews led to three main themes and nine subthemes. The three main themes were: 1. Special participant groups; 2. Organization and process; and 3. Effects. Figure 5. Shows the themes from the focus group interviews of LACP instructors.

#### 3.3.1. Special Participant Groups

*Emergency Medical Service*

The participation of EMS personnel was challenging as some of them have special needs and preferences. Some of them do not think that palliative care is a relevant topic for them. On the other hand, most EMS personnel engaged actively in the group discussions.

*Although a number of EMS personnel would prefer another topic for their continuing education many were very engaged in the group discussions.*

The LACP offers first contact with the topics of hospice care and reflection of one’s own attitude. An introduction to these topics can be beneficial for contact with relatives after distressing jobs and may improve empathic behaviour in emergency situations.

*In the end the day was very personal and emotional for everyone which was positive. However the written feedback was in a number of cases in contrast to the experiences from the course. I was astonished how positive the contact during the course was, although many wrote that the course has no positive influence on their daily work in the EMS service.*

*Integration support for disabled people*

Participants from integration support for disabled people showed great interest in the LACP and the topics of hospice care and palliative care. Many of them felt empowered and motivated by the LACP to handle ethical challenges and to care for disabled people in the last phase of life.

*I have made the experience that people from integration support for disabled people appreciated the course very much.*

During the discussions, the differences between claims and reality in institutions such as nursing homes and disabled facilities became obvious.

*A great difference between the wishes of the carers to accompany dying people in an appropriate way and the reality of that is possible in institutions became visible.*

*Managerial staff*

The support of managerial staff in palliative care education is important in order to promote positive attitudes in the institutions. Open communication between management and staff can enable open discussions and reflection about challenges and common values in the care for dying people. The participants’ examples were often about ethical conflicts in decision-making in emergency situations.

*Some managers demand the employees to sign a statement that they will resuscitate everyone who is not breathing anymore. This leads to ethical conflicts for the carers.*

Some participants want managerial staff to participate in the LACP. Others state that the participation of managers could hamper open discussion in the groups.

*The dynamic does change if managerial staff are present or not.*

*Some managers who participated in the course were not open for a change of perspective and emphasized that everything was perfect in their own institution. That complicated the group discussions.*

#### 3.3.2. Organization and Process

*Recruitment and information*

The experiences of the instructors show that personal contact helps to raise interest and to implement the LACP. The courses can be included in companies’ corporate health management.

*Our experience show that personal contacts are needed in the beginning to start a pyramid scheme to reach a broader group of people. The LACP can be integrated in the companies’ corporate health management which can improve the satisfaction of the employees in the workplace.*

Many institutions had difficulties sending their employees to the course due to shortages of personnel.

*There were concerns wether it would be possible to enable enough employees to participate the course if it was offered as in-house education.*

The cooperation of different institutions can be a measure for organizing the courses and recruiting enough participants.

*Usually the institutions are open to cooperating with other institutions to offer courses together. This enables a number of nursing homes from one region to organize the course together.*

*Mix of professions*

The participation of people with different professional backgrounds is positive as participants can share different perspectives and experiences. This can support the exchange between the participants, is very well appreciated and improves both the change in perspective and the learning process.

*In courses with different professions such as nursing, physicians, paramedics and social workers the participants learn from each other and develop an understanding for the perspectives of the different professions.*

This mix of professions supports the understanding for each other and is the basis for good cooperation for the care for seriously ill and dying people.

*The exchange with colleagues from different fields leads to an increased understanding of the participants’ own role and the challenges experienced by others—this is a helpful change of perspective.*

Some instructors would love more physicians as participants in the LACP. This underlines the importance of the mix of professions among the course participants.

*The nurses want all physicians and consultants to participate in the LACP. My most impressive experience was that a female physician recognized that she in her medical practice had not taken the needs of dying patients into account until now. She suggested that all physicians should participate in the LACP. I really liked that.*

*Course content and process*

Topic communication, care for the dying person and their relatives, and ethical aspects were highly appreciated by the participants and offer valuable help for daily practice. With regard to communication, the participants would like to get concrete suggestions on what to say.

*Many participants want a kind of guideline for the communication of imminent dying and conversations with relatives.*

Some participants want more concrete information about clinical signs of imminent death.

*In every course the question was brought up how to recognize that a person is dying. It was a reoccurring wish of many participants.*

Many participants found that the LACP was too long for one day.

*A lot of feedbacks stated that the LACP was valuable and highly appreciated. Some participants would like to see a shorter version.*

#### 3.3.3. Effects

*Change in perspective*

The change in perspective is a central part of the course that enables the participants to learn about and to understand the perspectives of both the other professions and the seriously ill person.

*In some courses the participants were able to change their perspective in a way that enabled them to understand the others’ perspectives without the need for other aids.*

The methods used in the LACP enabled the participants to reflect on frequently used ways of thinking and to explore new ways to care for dying people and their relatives. For many participants, changing their perspective is challenging, but it is part of the learning success.

*The group discussions were rated as very good and led to a lively exchange. Many participants found it challenging to act in the role of another profession in a group discussion.*

*Confidence and competence*

The participants gained more confidence in their own assessment and their use of empathy through group discussions and the case story.

*The LACP has made the participants more self secure. They have learned to express their own judgement and to become able to speak about it.*

To be able to speak about their own judgements and perspectives supports the participants in their professional role in order to be an advocate for the patients’ and relatives’ needs.

*The participants mentioned that they felt strengthened and more competent to act in their daily work through participation in the LACP.*

*Reflection of attitudes and ethical dilemmas*

The reflection on ethical aspects and one’s own attitudes is an important part of the course. The participants are encouraged to reflect on their own values and attitudes in connection to care for dying people and to reflect on ethical questions from their daily work.

*The nurses stated in their evaluation questionnaires how important the encounter with ethical dilemmas was and appreciated the possibility to reflect on their own values.*

This reflection does not only support personal growth but also supports the ability to make decisions in complex ethical situations. Some participants reported a change in their views through the course.

*The reflection on their own values and attitudes is for many participants a moving experience that encourages them to think new and to rethink.*

The reflection led to an increased awareness of ethical challenges in their daily work for some participants. This also led to a focus on existing differences between wishes for care and the reality.

*In the courses it became obvious that there is a big difference between the nurses’ wishes to provide appropriate care for dying people and the reality in institutions.*

Many participants found the reflection and discussion of ethical challenges very important and helpful. On the other hand, some participants felt that this was a burden. Interestingly some paramedics found the confrontation with their own mortality difficult.

*Some paramedics reported that they left the course because they found the intensive confrontation with their own morality and the ethical challenges difficult to keep up with.*

### 3.4. Additional Comments on the Experiences from the Implementation Process in Schleswig-Holstein

The first LACP pilot courses were started during the COVID-19 pandemic and held as online courses [7]. As the results were very positive and encouraging, the implementation in the federal state of Schleswig-Holstein and the current study were done between July 2021 and July 2024. The experiences of the project and research team showed that the staff shortage in health and social care was a major barrier for the implementation of LACPs. As written, a number of courses had to be cancelled due to a lack of staff to keep up the clinical work. This led to the cancellation of planned LACPs on short notice. Online courses might have been helpful to include participants from different areas but online LACPs were not as welcomed as during the pandemic. In addition, an online course would not have solved the problem of staff shortages. As shown above, the vast majority of people prefer courses in person.

## 4. Discussion

The most important results from the current study were: the LACPs were well accepted by different organizations and participants from different professions; LACPs were feasible in different places and with a different mix of professional audiences; the participants welcome the mix of professions and the opportunity for interprofessional exchange and the possibility for a change in perspectives. Thus, a mix of professions is an important aim for future courses. However, it was often complicated to organize the LACP with a mix of professions, especially if the course was held as an in-house course, e.g., in a long-term care facility or an emergency medical service facility. The participation of physicians was highly appreciated by many participants but in the data from the current study, only few physicians did participate in the LACP.

The biggest challenge during the project period was the existing staff shortage in the healthcare service and social service in the whole country during the study period. Due to that, a number of planned courses could not be held because personnel were not able to attend as they had to work. Thus, a major barrier for the wide implementation of LACPs is staff shortage in healthcare and social services. On the other hand, the LACP may contribute to improving the professionals’ satisfaction with their work as most feel empowered to handle end-of-life care and ethical challenges better. This may also contribute to preventing burnout and moral injury through reflection and discussion of ethical challenges associated with their daily work. Moral injury has been described as being connected to working in palliative care and other areas of healthcare including the emergency medical service [20,21,22]. As witnessing suffering and death can cause moral distress [23] and emotional exhaustion, it is highly relevant to talk about these situations and to address the ethical aspects associated with caring for palliative patients for all these groups. It has been shown that educational programmes can help to retain talented nursing personnel [24].

Most participants prefer LACPs in person instead of online courses. This could be a post-COVID pandemic effect as many people may be tired of online meetings and wish to meet and learn in a classroom setting.

The informants from our study have provided important suggestions for the revision of the LACP incuding course contents, methods, practical training and preferences for the length of the course. One important aspect is the suggestion of special modules or variations in used patient cases that can be used to suit the needs of different audiences, e.g., EMS personnel or professionals from integration support for disabled people. A working group of the German NGO Letzte Hilfe Deutschland gGmbH is currently working on a revision of the course curriculum of the LACP based on the scientific results and experiences of the instructors. Overall the results support the acceptance and feasibility of the LACP in healthcare and social services. A wider implementation seems useful and concepts for nationwide implementation are being discussed at present.

Several authors have stated that death literacy and public awareness of palliative care are low in society [25,26,27,28,29,30]. Unfortunately, healthcare professionals also have a “blurred conceptual understanding of palliative care” and differing interpretations of palliative care [31]. This can make it difficult to identify and refer patients in need of palliative care. Interprofessional communication and integration of education on palliative care in different levels of care are thus needed [31]. Interprofessional learning in the field of palliative care provides positive learning experiences for the participants, although it is a challenge to meet the learning needs of the different professions [32]. These results are similar to the findings from the current study that show that most participants value interprofessional education and the possibility to reflect on the views and perspectives of the different professions involved in palliative care provision in the community. One opportunity to address different needs of the participants could be the inclusion of a number of additional topics that the participants can choose from, e.g., acute death, suicide or more in-depth information on topics that are of special interest for the actual participant group, as previous research on LACPs for the public has shown that 9.4% of participants were nurses or physicians indicating an interest to learn more about dying, death, grief and palliative care in health care professionals [3]. One reason for the wish of medical professionals to learn more about dying, death and palliative care could be the lack of these topics in the professional education of these groups [3].

The use of the storyline method and the case discussions in groups were very much appreciated as an option for learning and reflecting on practices and ethical aspects of daily work by the participants from our study. According to the literature, the storyline method may be useful to cultivate empathy in undergraduate medical students [10].

The need for palliative care and end-of-life care in the community will increase in the coming years. Therefore, both the public and the healthcare providers in all communities have to engage with care for seriously ill and dying people. The LACP is one option for professionals to learn the basics of palliative care and to discuss dying, death and grief with others. The interdiscisplinary learning may contribute to a better understanding of different attitudes and professional roles and hopefully to better cooperation in the future.

### 4.1. Strengths and Limitations

The mixed-methods approach with a combination of quantitative and qualitative data from questionnaires for LACP participants and qualitative data from focus group interviews of LACP instructors can be seen as a strength. This source triangulation established a richer description of the experiences with the LACP. A limitation of the study is the fact that there were just a few participants from some professions, e.g., physicians or physiotherapists. As the data collection of the study took place from July 2021 to July 2024, social and contextual factors such as the COVID pandemic could probably have affected the study results during this period. Another limitation is the geographical restriction to the German federal state of Schleswig-Holstein. This fact was based on the funding from the government of the federal state. Although one can assume that the results might be similar in other parts of Germany, a nationwide study from all parts of Germany would be interesting. As the response rate of the LACP participants was 93%, the results are representative for the participant group formed of professionals from healthcare and social care in Schleswig-Holstein.

### 4.2. Practice Implications and Future Research Directions

Bringing death back into life has been suggested by the Lancet Commission of Death and Dying [33]. This can be achieved through a combination of LACs for the public and LACPs for professionals from healthcare and social care. LACs and LACPs can enhance the public debate on dying, death and grief and palliative care. Both course concepts could contribute to improving the awareness for palliative care throughout society and to encouraging citizens to cooperate with professionals in end-of-life care in the community.

Future research is needed on the effects of LACs and LACPs on palliative care and end-of-life care provision in the community and the number of home deaths for those who want to die at home. Our study showed differences between the participant groups, especially for EMS staff and staff from integration support for disabled people. These two groups need further attention and research on their special needs. At present, a study on the views, needs and wishes of EMS staff for the LACP is ongoing. A similar study for staff from integration support for disabled people is in the planning phase. In addition, the effect of the course concepts on attitude and behaviour in the months and years after course participation should be assessed. Another important aspect would be to look at the contribution of LACs and LACPs to establishing sustainable compassionate communities.

## 5. Conclusions

The results from our study show that the LACP is very well accepted by most participants from different professions. Further research on the long-term effects of LACPs on the participants’ knowledge, attitude and behaviour is needed. A wider implementation of LACPs in Germany seems to be useful to improve awareness and basic knowledge of palliative care throughout healthcare and social services in order to contribute to improved palliative care and end-of-life care in the community.

## Figures and Tables

**Table 1:** Modules and contents of the Last Aid Course Professional (LACP). One teaching hour = 45 min.

Module Nr.	Teaching Hours (Minutes)	Main Topic	Course Content
Module 1	2 (90 min)	Dying as a normal part of life	Welcome and introductions First Aid and Last Aid Networks of support Caring communities The process of dying Group discussion/practice: Self-awareness exercise
Module 2	2.5 (112.5 min)	Planning ahead	Making decisions Medical and ethical aspects Dignity Advance care planning Advance directive Medical power of attorney Group discussion/practice: Case discussions on planning ahead and treatment options
Module 3	2.5 (112.5 min)	Relieving suffering and symptom management	Typical problems and symptoms Total pain model Caring/relieving suffering Nutrition at the end of life Care at the end of life Group discussion/practice: Case discussions on symptom management Practice mouth care
Module 4	3 (135 min)	Final goodbyes	Five-finger rule of end-of-life care Farewell rituals Funerals and various forms of burials Grief and ways of grieving Self-care tools Questions, comments and evaluation Group discussion/practice: Case discussions on the dying phase

**Table 3:** Profession/role of the LACP participants (*n* = 394).

Profession	Number
Nursing	113
Emergency medical service	96
Elderly care	45
Educator/special education nurse	41
Professional caregivers	11
Medical assistant	10
Ergotherapist	8
Physician	8
Assistant	3
Administration/information technology	3
Special education teacher	3
Janitor	2
Midwife	2
Physiotherapist	2
Pharmacist	1
Volunteer	1
Other	24
No information	21

**Table 4:** Assessment of the LACP modules by the participants (*n* = 394).

Module and Topic	Assessment						
	1	2	3	4	5	6	No Information
1. Dying as a normal part of life	161	177	34	0	1	0	21
	41%	45%	8.7%	0%	0.3%	0%	5%
2. Planning ahead	143	180	48	2	0	0	21
	36.3%	46%	12.2%	0.5%	0%	0%	5%
3. Relieving suffering and symptom management	179	158	36	2	0	0	21
	45.5%	40%	9%	0.5%	0%	0%	5%
4. Final goodbyes	167	152	42	7	0	0	26
	42%	38%	11%	2%	0%	0%	7%
Assessment of the whole course	158	156	36	0	0	0	44
	40%	40%	9%	0%	0%	0%	11%

Assessment: 1 = very good, 2 = good, 3 = satisfactory, 4 = sufficient, 5 = insufficient, 6 = poor.

**Table 5:** Questions about the participants’ impressions (*n* = 394).

Statement from the Questionnaire Answered with Yes	All Participants *n* = 394	Emergency Medical Service Personnel *n* = 96	Educator/Special Education Nurse *n* = 41
The course is helpful for all professions within healthcare/care	331	53	41
	84%	55%	100%
I have learned many new things	273	55	37
	69%	57%	90%
The contents have been taught in an understandable way	390	95	40
	99%	99%	98%
I will recommend the course to others	355	72	39
	90%	75%	95%

## Data Availability

The original data from the questionnaires are stored on paper questionnaires and are therefore not openly accessible. Most of the original data presented in this study are included in the article material. Further inquiries can be directed to the corresponding author.

## Abbreviations

The following abbreviations are used in this manuscript:

ACP	Advance Care Planning
EMS	Emergency Medical Service
LAC	Last Aid Course
LACP	Last Aid Course Professional
LARGI	Last Aid Research Group International
OLACP	Online Last Aid Course Professional
PPCE	Public Palliative Care Education
